# De novo analysis of transcriptome reveals genes associated with leaf abscission in sugarcane (*Saccharum officinarum L.*)

**DOI:** 10.1186/s12864-016-2552-2

**Published:** 2016-03-05

**Authors:** Ming Li, Zhaoxu Liang, Yuan Zeng, Yan Jing, Kaichao Wu, Jun Liang, Shanshan He, Guanyu Wang, Zhanghong Mo, Fang Tan, Song Li, Lunwang Wang

**Affiliations:** Sugarcane Research Institute, Guangxi Academy of Agricultural Sciences, Nanning, 530007 P.R. China; Guangxi Academy of Agricultural Sciences, Nanning, 530007 P.R. China

**Keywords:** Leaf abscission, Leaf shedding, Sugarcane, Plant-pathogen interaction, Abscisic acid, ABA, *Saccharum officinarum*, Transcriptome

## Abstract

**Background:**

Sugarcane (*Saccharum officinarum L*.) is an important sugar crop which belongs to the grass family and can be used for fuel ethanol production. The growing demands for sugar and biofuel is asking for breeding a sugarcane variety that can shed their leaves during the maturity time due to the increasing cost on sugarcane harvest.

**Results:**

To determine leaf abscission related genes in sugarcane, we generated 524,328,950 paired reads with RNA-Seq and profiled the transcriptome of new born leaves of leaf abscission sugarcane varieties (Q1 and T) and leaf packaging sugarcane varieties (Q2 and B). Initially, 275,018 transcripts were assembled with N50 of 1,177 bp. Next, the transcriptome was annotated by mapping them to NR, UniProtKB/Swiss-Prot, Gene Ontology and KEGG pathway databases. Further, we used TransDecoder and Trinotate to obtain the likely proteins and annotate them in terms of known proteins, protein domains, signal peptides, transmembrane regions and rRNA transcripts. Different expression analysis showed 1,202 transcripts were up regulated in leaf abscission sugarcane varieties, relatively to the leaf packaging sugarcane varieties. Functional analysis told us 62, 38 and 10 upregulated transcripts were involved in plant-pathogen interaction, response to stress and abscisic acid associated pathways, respectively. The upregulation of transcripts encoding 4 disease resistance proteins (RPM1, RPP13, RGA2, and RGA4), 6 ABC transporter G family members and 16 transcription factors including WRK33 and heat stress transcription factors indicate they may be used as candidate genes for sugarcane breeding. The expression levels of transcripts were validated by qRT-PCR. In addition, we characterized 3,722 SNPs between leaf abscission and leaf packaging sugarcane plants.

**Conclusion:**

Our results showed leaf abscission associated genes in sugarcane during the maturity period. The output of this study provides a valuable resource for future genetic and genomic studies in sugarcane.

**Electronic supplementary material:**

The online version of this article (doi:10.1186/s12864-016-2552-2) contains supplementary material, which is available to authorized users.

## Background

Sugarcane (*Saccharum officinarum L.*) is an important sugar crop, which is widely grown in the tropical and subtropical areas [1]. The total sugarcane acreage all over the world is more than 200 million hectares, yielding over 13 billion tons of sugar per year [2]. Apart of this, sugarcane is an ideal non-food biomass crop which can produce two kinds of biofuel: ethanol and high biomass raw sugar [3]. In China, sugarcane is mainly distributed in the provinces of Guangxi, Yunnan, Guangdong and Hainan, of them Guangxi province produces more than 60 % of the total sugar per year. The sugarcane harvest mainly relies on hand-cut in several countries like China, large-scale mechanized harvest is less than 15 % of the total amount, and the cost of sugarcane is increasing. Hence, it is important to study the mechanism of leaf abscission in sugarcane and breed sugarcane varieties which can shed their leaves during the maturity time.

Abscission is the programmed developmental process by which some of the organs such as leaves, flowers, or fruits are shed during the life of a plant [4]. It occurs within a specific tissue, called abscission zone (AZ), which is formed at the base of the petiole [5]. The abscission process can be divided into four major steps [6]: development of the AZ tissue (step 1), acquisition of competence to respond to abscission-promoting signaling (step 2), activation of abscission (step 3), and post abscission trans-differentiation (step 4). Steps 2 and 3 have been extensively investigated, by transcriptome analyses, in the gene expression changes during the shedding of AZs tissues, such as leaves, flowers and fruits [7–12]. It is interesting that different plant species and different organs share a majority of genes involved in steps 2 and 3, including genes involved in ethylene and auxin biosynthesis and signal transduction, cell wall modification and various stress responses [12].

Although some genes have been found to regulate the development of AZs tissues (step 1), they are different across species and organs. In *Arabidopsis*, the formation of seed AZs is regulated by the MADS-box transcription factor (TF) gene *SEEDSTICK (STK)* and the bHLH transcription factor gene *HECATE3 (HEC3)* [13, 14], but the formation of *Arabidopsis* floral organ AZs is regulated by *BLADE-ON-PETIOLE1 (BOP1)* and *BOP2* which encode BTB/POZ domain and Ankyrin repeat containing NPR1-like proteins [5]. In tomato, MACROCALYX and JOINTLESS containing a MADS-box controls the fruit and flower AZ development [12, 15]. In rice, several genes and their products have been reported to regulate the pedicel AZ formation for seed shattering, including *qSH1* (a major chromosome 1 quantitative trait locus for seed shattering, encoding a BELL-type homeobox TF) [16], *SH4* (a major chromosome 4 seed shattering quantitative trait locus, encoding a MYB3 DNA-binding domain containing protein) [17], *HATTERING ABORTION1* (*SHAT1*, encoding an AP2 family TF) [18], the rice *SHATTERING1* homologue (*OsSH1*, encoding a YAB family TF) [19], and *CTD phosphatase-like protein1* (*OsCPL1*) [20]. Overall, AZ development associated genes vary from one to another plant species. In sugarcane, genes controlling leaf AZ development and leaf abscission are still unknown.

RNA-Seq, a next generation sequencing technology, is used for profiling gene expression and plant breeding programs in many plants including rice [21], maize [22] and millet [23]. The genome sequence of sugarcane is not available currently, so transcriptome studies in sugarcane have been proposed, in progress or accomplished in different countries like South Africa [24, 25], Australia2 [26, 27] and USA [28]. Here, we performed an RNA-Seq study to profile the gene expression of new born leaf tissues using the HiSeq 2000 platform. Differential expression analysis revealed 1,202 transcripts upregulated in leaf abscission sugarcane plants (LASP) which can shed their leaves during the maturity time, compared to the leaf packaging sugarcane plants (LPSP) which are packed by the leaves during the maturity time. Functional analysis showed the upregulated transcripts in LASP were enriched in “plant-pathogen interaction”, “response to stress” and abscisic acid (ABA) associated pathways. Due to their up regulation, we assumed these transcripts may involve in the processes of AZ development and leaf abscission in sugarcane. This is the first time to study the genes associated with AZ development and leaf abscission in sugarcane. Our results will provide a valuable resource for understanding the mechanism of leaf abscission in sugarcane and will contribute to the researchers in the field of sugarcane breeding.

## Results and discussion

### Deep sequencing and de novo assembly

**Fig. 1:**
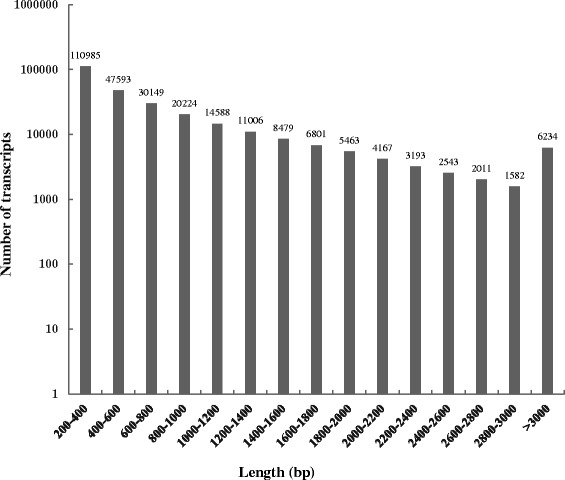
Length distribution of sugarcane leaf transcriptome assembled by Trinity

In order to understand the mechanism of leaf abscission in sugarcane, six transcriptome libraries for two parent sugarcane varieties (Q1 and Q2), two F1 generation sugarcane varieties (T1 and T2), which can shed their leaves during the maturity time, and another two F1 generation sugarcanes (B1 and B2), which are packed by the leaves during the maturity, were constructed and sequenced. As shown in Table 1, by using the HiSeq 2000 platform we generated a total of 524,328,950 paired raw reads for six libraries. The Q20 values of six libraries were from 97.58 % to 97.96 %. After removing low quality reads and reads with adaptors, we obtained ~76.9 M, ~83.2 M, ~81.4 M, ~77.2 M, ~78.6 M and ~80.9 M clean reads for B1, B2, Q1, Q2, T1 and T2, respectively. As the estimated genome sizes of *S. officinarum* accessions ranged from 7.50 to 8.55 Gb with an average size of 7.88 Gb [29], our data equivalent to ~10-fold coverage of the sugarcane genome sequences. The clean reads (478.1 M) were then used for de novo assembly analysis by Trinity software [30], resulting a total of 275,018 unique transcripts corresponding to 164,803 genes. The GC percentage of the assembled transcripts was 48.23 %, the N50 statistic was 1,177 which represented at least 50 % of the sum of the lengths of all contigs include contigs that were at least 1,177 bp long, and the distribution of contig length can be seen in Fig. 1. Approximately 40 % of the assembled contigs were 200 ~ 400 bp in length, however, we obtained 67,238 contigs longer than 1,000 bp. The longest contig was 15,553 bp in length and a total of 6,234 contigs were longer than 3,000 bp. The number of total assembled bases was 215,019,578, which meant about 215 M size of mRNA sequences were characterized in this study.

**Table 1:** Overview of transcriptome sequencing and de novo assembl results

	B1	B2	Q1	Q2	T1	T2
Raw reads	83,879,424	92,222,556	89,370,426	89,370,426	85,447,812	88,421,042
Q20 percentage	97.96 %	97.58 %	97.77 %	97.66 %	97.94 %	97.86 %
Clean reads	76,869,838	83,161,522	81,439,136	77,208,082	78,574,934	80,853,072
Total reads	478,106,584					
Total trinity ‘genes’	164,803					
Total trinity transcripts	275,018					
Percent GC	48.23 %					
ContigN10	3,155					
ContigN50	1,177					
Total assembled bases	215,019,578

### Functional annotation of assembled transcripts

**Fig. 2:**
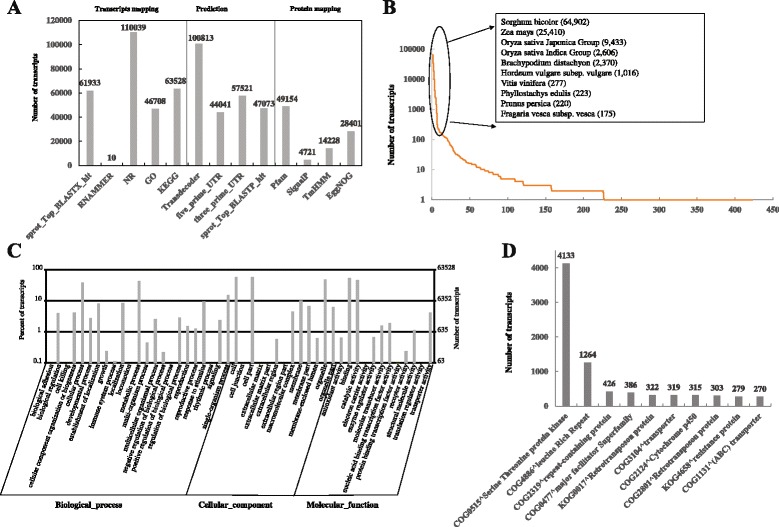
Functional annotation for the assembled transcripts. **a** The numbers of transcripts or putative proteins mapping to public databases or annotated based on the conservation. **b** Distribution of species aligned by the assembled transcripts. **c** Gene Ontology annotation for the assembled transcriptome. **d** Top 10 COGs annotations

To understand the features and functions of the assembled transcripts, we annotated the assembled transcripts by mapping them to several public databases, like NCBI non-redundant (NR), UniProtKB/Swiss-Prot, GO and KEGG pathway. The numbers of transcripts aligned to each database can be seen in Fig. 2a. A total of 110,486 (40.17 %) transcripts were annotated, of which 110,039 (40.01 %) were mapping to NR database under the e-value of 1 × 10^−5^. As expected, in the NR mapping results we found 64,902 (58.98 %), 25,410 (23.09 %), 9,433 (8.57 %) and 2,606 (2.37 %) assembled transcripts were aligned to *Sorghum bicolor*, *Zea mays*, *Oryza sativa Japonica Group* and *Oryza sativa Indica Group*, respectively (Fig. 2b, Additional file 1). Due to the unavailability of sugarcane genome and gene sequences in public databases, we found only 550 transcripts were mapping to *Saccharum* species. GO analysis is an international standardized gene function classification system that provides a controlled vocabulary to facilitate high-quality functional gene annotation [31, 32]. GO term distribution (Fig. 2c) of the assembled transcripts showed the “cellular process” and “metabolic process” were two most abundantly represented with 24,907 (53.32 %) and 27,955 (59.85 %) transcripts, respectively. In the “cellular components” ontology transcripts were mainly distributed in “cell” (36,954, 79.12 %), “cell part” (36,954, 79.12 %) and “organelle” (30,693, 65.71 %). GO analysis also showed 33,849 (72.47 %) transcripts had “binding” function and 30,060 (64.36 %) with “catalytic activity” function in “molecular function” ontology. Detailed information can be found in Additional file 2. In addition, some transcripts (59.83 %) showed no similarity to any known protein database, there were probabilities that they were putative long noncoding RNAs or novel genes in sugarcane [33]. More experiments are required to characterize them [34, 35].

Then, we extracted likely coding sequences from the assembled transcripts using TransDecoder. In total, 100,813 likely protein sequences, 44,041 5’-prime-UTRs and 57,521 3’-prime-UTRs (Fig. 2a) were obtained. By using the Trinotate pipeline likely protein sequences were annotated in terms of known proteins, protein domains, signal peptides, transmembrane regions and rRNA transcripts (Fig. 2a). It showed 47,073 (46.69 %) of total likely protein sequences were aligned to UniProtKB/Swiss-Prot under e-values of 1e-5 by BLASTX, 49,154 (48.76 %) protein sequences were characterized to have protein domains of Pfam, 4,721 (4.68 %) potential signal proteins were identified by SignalP [36] and 14,228 (14.11 %) proteins were found with high similarity to membrane proteins by TMHMM Sever v2.0 [37]. In addition, 10 transcripts were identified as ribosomal rRNAs, 28,401 protein sequences were aligned to EggNOG database (v4.1) [38] resulting 1,363 COGs, 43 KOGs, 21 euNOGs and 333 NOGs. According to the numbers of transcripts mapping to the EggNOG groups, top 10 were shown in Fig. 2d. Likely protein sequences of sugarcane leaves were annotated with “threonine protein kinase” (4,133 transcripts) which had more than three times of the second “leucine rich repeat” (1264 transcripts). The threonine protein kinase has been reported to play a key role in the regulation of cell proliferation, cell differentiation and cell death [39, 40]. These annotations for the assembled transcripts including gene and protein description, putative conserved domains and potential biological pathways provided a valuable resource for subsequent investigation of specific biological processes, functions and pathways in cell death and sugarcane leaf shedding.

### Transcriptome profile

**Fig. 3:**
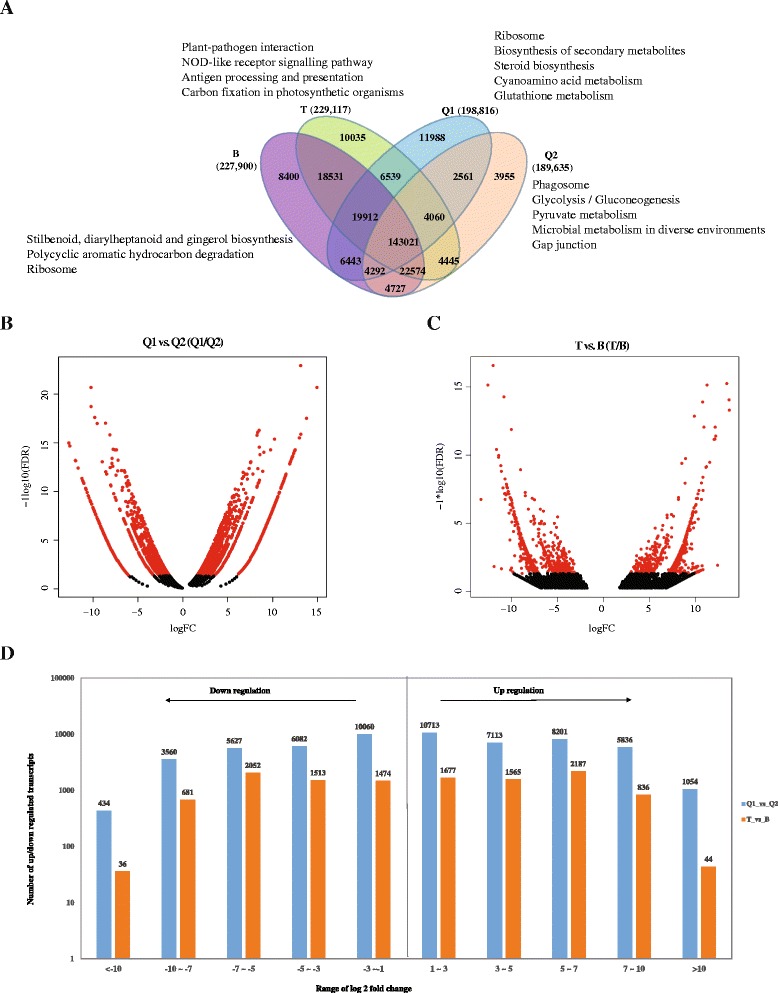
Transcriptome profile and different expression. **a** Venn diagram of transcripts detected in Q1, Q2, T and B. **b** Volcano plot of differentially expressed transcripts between Q1 and Q2. **c** Volcano plot of differentially expressed transcripts between T and B. **d** Numbers of up- and down-regulated transcripts identified in Q1 vs. Q2 and T vs. B

We next aligned the clean reads of all six samples to the assembled transcripts using Bowtie2 [41] and estimated the abundance of each transcript using RSEM (RNA-Seq by Expectation-Maximization) [42]. According to the phenotypes of F1 generation sugarcane varieties, B1 and B2 were considered as B, T1 and T2 samples were considered as T in this study. As shown in Fig. 3a, we detected a total of 198,816, 189,635, 229,117 and 227,900 transcripts in Q1, Q2, T and B, respectively. There were 143,021 transcripts common to all the samples. It is notable that 11,988, 3955, 10,035 and 8400 transcripts were exclusively detected in Q1, Q2, T and B, respectively. KEGG pathway analysis showed specifically expressed transcripts were enriched in different biological pathways. For example, transcripts unique to B were mainly enriched in “ribosome” (ko03011, 34 transcripts), “polycyclic aromatic hydrocarbon degradation” (ko00624, 12 transcripts), and “stilbenoid, diarylheptanoid and gingerol biosynthesis” (ko00945, 15 transcripts). Transcripts exclusively detected in T might involve in “plant-pathogen interaction” (ko04626, 94 transcripts), “NOD-like receptor signaling pathway” (ko04621, 12 transcripts), and “antigen processing and presentation” (ko04612, 16 transcripts). Different KEGG pathways for transcripts detected only in B and T indicated they may have differences on their genetic information and phenotypes.

### Differentially expressed transcripts

We next used edgeR [43] to identify differentially expressed transcripts in LASP (Q1 and T), compared to LPSP (Q2 and B). As described in Material and Methods, we filtered the transcripts by their fold changes (>2) and *p*-values (<0.05). By using this critical, differentially expressed transcripts were shown in red in the volcano plots of Fig. 3b and c. In total, we detected 32,917 transcripts upregulated and 25,764 transcripts down regulated in Q1 sugarcane in comparison of Q2 sugarcane (Fig. 3b), and 6,309 upregulated and 5,757 down regulated transcripts in T sugarcane relatively to B sugarcane (Fig. 3c). The numbers of up and down regulated transcripts in different ranges can be found in Fig. 3d. Notably, 1,054 and 44 transcripts were upregulated very significantly (log2FC >10) in Q1 and T in comparison of Q1 and B, respectively. In addition, we found Q1 and T shared a total of 1,202 upregulated and 953 down regulated transcripts (Additional file 3). GO analysis of the commonly upregulated transcripts (Table 2) showed 38, 35 and 15 transcripts were enriched in “response to stress”, “transition metal ion binding” and “cellular protein modification process”, respectively. KEGG pathway analysis (Table 3) showed commonly upregulated transcripts were enriched in the pathways of “plant-pathogen interaction”, “one carbon pool by folate” and “diterpenoid biosynthesis”.

**Table 2:** GO analysis for the commonly up regulated transcripts

Gene Ontology	GO_item	Transcript_number	*P*-value	Q-value
Cellular component	intracellular part	9	2.43E-02	4.38E-01
Molecular function	transition metal ion binding	35	1.02E-05	8.43E-04
	iron ion binding	9	1.41E-05	1.17E-03
	amine transmembrane transporter activity	1	1.08E-04	8.96E-03
	nucleic acid binding transcription factor activity	6	6.72E-04	5.58E-02
Biological process	cellular homeostasis	3	5.30E-13	4.19E-11
	organic substance transport	5	1.05E-09	8.32E-08
	establishment of localization	2	3.04E-07	2.40E-05
	response to stress	38	1.08E-06	8.50E-05
	amino acid transport	1	7.57E-06	5.98E-04
	DNA metabolic process	5	1.21E-05	9.53E-04
	translation	2	1.16E-04	9.14E-03
	purine nucleoside monophosphate biosynthetic process	1	3.29E-04	2.60E-02
	cell morphogenesis	1	3.62E-04	2.86E-02
	cellular protein modification process	15	7.43E-04	5.87E-02
	transport	3	1.00E-03	7.91E-02
	proteasomal ubiquitin-dependent protein catabolic process	2	1.59E-03	1.26E-01
	cell wall biogenesis	2	1.94E-03	1.54E-01
	vitamin K biosynthetic process	1	4.69E-03	3.71E-01
	primary metabolic process	4	5.85E-03	4.62E-01
	one-carbon metabolic process	7	6.35E-03	5.02E-01
	branched-chain amino acid metabolic process	2	6.99E-03	5.52E-01
	response to metal ion	4	7.13E-03	5.63E-01
	polysaccharide metabolic process	1	7.30E-03	5.76E-01
	glucose catabolic process	3	1.08E-02	8.55E-01

**Table 3:** Significant KEGG pathways of commonly up-regulated transcripts in Q1 vs. Q2 and T vs. B

Pathway	ID	Transcritps_number	*P*-value	Q-value
Plant-pathogen interaction	ko04626	62	1.49E-09	2.68E-07
One carbon pool by folate	ko00670	20	5.73E-06	1.03E-03
Diterpenoid biosynthesis	ko00904	8	4.39E-05	7.90E-03
Homologous recombination	ko03440	20	1.18E-04	2.13E-02
Epstein-Barr virus infection	ko05169	24	3.37E-04	6.07E-02
Cytosolic DNA-sensing pathway	ko04623	17	4.01E-04	7.22E-02
Two-component system	ko02020	18	5.82E-04	1.05E-01
Nitrogen metabolism	ko00910	10	9.44E-04	1.70E-01
Drug metabolism - cytochrome P450	ko00982	10	1.21E-03	2.17E-01
Histidine metabolism	ko00340	6	1.38E-03	2.49E-01
Chloroalkane and chloroalkene degradation	ko00625	9	1.99E-03	3.58E-01
RNA polymerase	ko03020	17	2.59E-03	4.65E-01
Metabolism of xenobiotics by cytochrome P450	ko00980	9	2.76E-03	4.97E-01
Naphthalene degradation	ko00626	7	4.81E-03	8.66E-01

We were surprised that Q1-up-regulated transcripts and T-up-regulated transcripts were enriched in different KEGG pathways (Additional file 4 and Additional file 5), indicating they might have different regulatory pathways of leaf abscission during the maturity time. Transcripts upregulated in Q1 (compared to Q2) were enriched in the auxin related pathways, like “flavonoid biosynthesis” (151 transcripts, *p*-value: 2.20E-16), “limonene and pinene degradation” (427 transcripts, *p*-value: 2.20E-16), “plant hormone signal transduction” (606 transcripts, *p*-value: 2.20E-16) and “phenylpropanoid biosynthesis” (343 transcripts, *p*-value: 2.20E-16). However, transcripts upregulated in T (compared to B) were enriched in the pathways of “plant-pathogen interaction” (210 transcripts, *p*-value: 2.20E-16), “one carbon pool by folate” (59 transcripts, *p*-value: 1.96E-11) and “homologous recombination” (64 transcripts, *p*-value: 7.24E-10). As we know, internal and environmental signals can influence and proceed the leaf senescence and death, including abiotic factors like drought, nutrient limitation, extreme temperature, and oxidative stress by UV-B (ultraviolet B) irradiation and ozone [44, 45]. It is inferred that leaf abscission of sugarcane is linked to at least one of these pathways.

### Transcripts associated with leaf abscission

The leaf abscission mechanism in sugarcane is still unknown, however, genes associated with hormonal regulation, stress and diseases has been studied to regulate the process of leaf abscission in other plants [46–49]. In this study, we analyzed the commonly upregulated transcripts in LASP in comparison of LPSP and found genes related to leaf abscission in sugarcane, which involved in plant-pathogen interaction, responses to stress and ABA-associated pathways.

### Plant-pathogen interactions

First, we analyzed the transcripts of “plant-pathogen interaction” because it was the most significant KEGG pathway of commonly upregulated transcripts. Unlike in animals, pathogen interactions often trigger programmed cell death in plants [50–52]. Among the commonly upregulated transcripts, 62 were annotated to regulate the pathway of plant-pathogen interaction. Table 4 showed their log 2 values of fold changes, ranging from 1.45 to 11.45 in Q1/Q2 and 1.35 to 11.12 in T/B. Of these 62 transcripts, 36 (58.06 %) transcripts were annotated to encode disease resistance proteins, including 7 RPM1 (resistance to *Pseudomonas syringae pv. Maculicola* 1), transcripts, 7 RPP13 (recognition of *Peronospora parasitica* 13) transcripts and 6 RPP13-like transcripts. Both RPM1 and RPP13 can trigger the plant defense process [53, 54]. In *Arabidopsis*, RPM1 acts through its interaction with RIN4 (RPM1-interacting protein 4), an essential regulator of plant defense, and triggers plant disease resistance when RIN4 is phosphorylated by AvrRpm1 [55, 56]. After infection of tomato yellow leaf curl virus, RPP13 is upregulated in a resistant tomato line [57]. In addition, a WRKY transcription factor called WRK33 was identified to regulate the plant-pathogen interaction. WRK33 can interact with the W box (5’-TTGAC[CT]-3’, an elicitor-responsive cis-acting element) [58] and function in ABA signaling [59]. In *Arabidopsis*, WRK33 is involved in regulation of the antagonistic relationship between defense pathways mediating responses to the bacterial pathogen *P. syringae* and the necrotrophic pathogen *B. cinerea* promoters [60]. The up regulation of transcripts encoding plant disease resistance proteins suggested that the defense system of sugarcane was activated by some reason and might contribute on shedding sugarcane leaves during the maturity time.

**Table 4:** Transcripts involved in the pathway of plant-pathogene interaction

Gene family	gene_id	log2FC(Q1/Q2)	*P*Value	log2FC(T/B)	*P*Value	Description
RX24L	c97780_g1_i3	3.228	#######	6.523	#######	Probable disease resistance protein RXW24L
	c105254_g1_i2	1.990	#######	2.181	#######	Calcium-dependent protein kinase isoform 11
DRL	c101464_g2_i5	2.348	#######	2.145	#######	Putative disease resistance protein At1g50180
	c77488_g1_i1	2.807	#######	6.112	#######	
	c93620_g2_i1	5.716	#######	5.264	#######	
	c97780_g3_i1	6.722	#######	5.438	#######	
	c142188_g1_i1	3.527	#######	4.753	#######	Probable disease resistance protein RDL6
ERECT	c105005_g2_i1	3.139	#######	1.797	#######	LRR receptor-like serine/threonine-protein kinase ERECTA
FB95	c82142_g1_i1	2.097	#######	7.978	#######	F-box protein At2g02240
FLS2	c104719_g1_i2	6.103	#######	2.347	#######	LRR receptor-like serine/threonine-protein kinase FLS2
HSP82	c108102_g3_i1	3.494	#######	2.543	#######	Heat shock protein 82
	c76241_g1_i1	2.373	#######	2.374	#######	
LRC40	c15793_g1_i1	5.998	#######	5.839	#######	Leucine-rich repeat-containing protein 40
LRK91	c99649_g1_i3	5.808	#######	5.880	#######	L-type lectin-domain containing receptor kinase IX.1
MPK5	c83394_g1_i1	4.061	#######	1.615	#######	Mitogen-activated protein kinase 5
R13L family	c105793_g1_i13	2.664	#######	2.311	#######	Putative disease resistance RPP13-like protein 2
	c105793_g1_i8	8.150	#######	4.113	#######	
	c105793_g1_i9	1.563	#######	7.504	#######	
	c67555_g1_i1	3.908	#######	2.860	#######	Putative disease resistance RPP13-like protein 3
	c101028_g2_i1	9.403	#######	8.190	#######	Disease resistance RPP13-like protein 4
	c101028_g2_i5	8.861	#######	6.967	#######	
R1B	c104414_g3_i1	4.078	#######	4.774	#######	Putative late blight resistance protein homolog R1B-12
	c82142_g2_i1	2.352	#######	6.412	#######	
	c97780_g2_i3	2.354	#######	6.259	#######	
	c105793_g1_i1	3.268	#######	1.792	#######	Putative late blight resistance protein homolog R1B-16
	c68709_g1_i1	3.122	#######	3.125	#######	
RGA	c91884_g5_i3	1.449	#######	2.760	#######	Putative disease resistance protein RGA3
	c102886_g1_i4	5.643	#######	2.903	#######	Putative disease resistance protein RGA4
	c91884_g5_i4	2.167	#######	2.247	#######	
RP8L3	c97780_g1_i1	7.405	#######	6.223	#######	Disease resistance RPP8-like protein 3
	c97780_g1_i5	5.565	#######	6.223	#######	
RPM1	c107814_g1_i1	2.025	#######	1.350	#######	Disease resistance protein RPM1
	c108644_g1_i2	2.759	#######	2.277	#######	
	c66665_g1_i1	9.403	#######	7.371	#######	
	c68216_g1_i1	9.485	#######	5.894	#######	
	c95380_g2_i1	8.265	#######	9.699	#######	
	c95380_g2_i2	8.438	#######	9.519	#######	
	c99461_g1_i2	2.318	#######	3.144	#######	
RPP	c104393_g1_i1	3.763	#######	3.659	#######	Disease resistance protein RPP13
	c104393_g1_i3	3.376	#######	5.385	#######	
	c104393_g1_i8	2.979	#######	3.708	#######	
	c104393_g1_i9	2.827	#######	2.926	#######	
	c107654_g2_i3	2.151	#######	2.678	#######	
	c89572_g4_i3	1.611	#######	3.859	#######	
	c97780_g5_i1	2.086	#######	5.392	#######	
RSLE2	c99739_g2_i1	2.603	#######	1.697	#######	Zinc finger BED domain-containing protein RICESLEEPER 2
	c99739_g2_i3	8.733	#######	3.065	#######	
	c99739_g2_i7	9.022	#######	2.909	#######	
SD25	c99065_g1_i9	6.315	#######	5.297	#######	G-type lectin S-receptor-like serine/threonine-protein kinase SD2-5
WAK1	c48856_g1_i1	11.447	#######	11.123	#######	Wall-associated receptor kinase 1
WRK33	c99739_g2_i8	6.689	#######	3.657	#######	Probable WRKY transcription factor 33
Y1571	c103804_g1_i1	6.863	#######	2.780	#######	Probable leucine-rich repeat receptor-like protein kinase At1g35710
	c103804_g1_i2	6.400	#######	2.506	#######	
Y3475	c103043_g1_i3	4.158	#######	3.636	#######	Probable LRR receptor-like serine/threonine-protein kinase At3g47570
	c104719_g1_i1	6.051	#######	2.048	#######	
Y4885	c103804_g1_i3	6.967	#######	3.236	#######	Probable LRR receptor-like serine/threonine-protein kinase At4g08850
	c105253_g3_i2	4.866	#######	2.290	#######	
gi|414591554|tpg|DAA42125.1|	c101028_g1_i1	7.789	#######	6.349	#######	TPA: hypothetical protein ZEAMMB73_852544 [Zea mays]
gi|242077232|ref|XP_002448552.1|	c65099_g1_i1	3.245	#######	2.407	#######	hypothetical protein SORBIDRAFT_06g028920 [Sorghum bicolor]
	c65099_g1_i2	2.978	#######	1.949	#######	
gi|242047620|ref|XP_002461556.1|	c75847_g1_i2	5.808	#######	6.270	#######	hypothetical protein SORBIDRAFT_02g004690 [Sorghum bicolor]
gi|115489000|ref|NP_001066987.1|	c91026_g3_i1	1.813	#######	5.859	#######	Os12g0553200 [Oryza sativa Japonica Group]

**Table 5:** Transcripts involved in the response of stress

Gene family	gene_id	logFC(Q1/Q2)	*P*Value	logFC(T/B)	*P*Value	Description
DRL4	c101464_g2_i5	2.348	2.01E-03	2.145	2.57E-02	Putative disease resistance protein At1g50180
	c77488_g1_i1	2.807	2.91E-03	6.112	3.75E-03	
	c97780_g3_i1	6.722	5.41E-11	5.438	4.32E-02	
DRL45	c142188_g1_i1	3.527	6.40E-05	4.753	1.21E-02	Probable disease resistance protein RDL6
ESAG8	c53232_g2_i1	1.943	1.03E-02	5.900	3.71E-02	Putative adenylate cyclase regulatory protein
PER1	c97626_g3_i1	5.639	2.59E-11	2.054	4.31E-02	Cationic peroxidase SPC4
	c98359_g1_i4	9.281	1.47E-08	4.586	1.56E-02	
PPD4	c108711_g1_i2	3.677	4.47E-07	2.058	4.54E-03	PsbP domain-containing protein 4, chloroplastic
R1B12	c97780_g2_i3	2.354	1.35E-02	6.259	2.52E-03	Putative late blight resistance protein homolog R1B-12
RGA2	c108711_g2_i1	4.177	2.28E-08	2.425	1.46E-03	Disease resistance protein RGA2
	c108711_g2_i2	3.511	1.23E-06	2.322	2.15E-03	
	c71260_g1_i1	12.593	7.40E-18	2.771	4.90E-02	
	c89143_g1_i1	8.593	7.45E-07	2.820	4.35E-02	
RGA4	c102886_g1_i4	5.643	1.63E-06	2.903	2.82E-02	Putative disease resistance protein RGA4
	c91884_g5_i4	2.167	1.72E-02	2.247	6.76E-03	
RPM1	c107814_g1_i1	2.025	2.98E-03	1.350	4.78E-02	Disease resistance protein RPM1
	c108644_g1_i2	2.759	2.86E-04	2.277	9.81E-03	
RPP13	c107654_g2_i3	2.151	2.26E-03	2.678	3.36E-03	Disease resistance protein RPP13
	c89572_g4_i3	1.611	3.32E-02	3.859	2.17E-03	
	c97780_g5_i1	2.086	1.55E-02	5.392	2.94E-02	
RPP8	c107654_g1_i3	2.435	1.44E-03	2.793	1.78E-02	Disease resistance protein RPP8
RSH3C	c74407_g1_i1	9.516	3.94E-09	3.288	1.93E-02	Probable GTP diphosphokinase RSH3, chloroplastic
gi|218187380|gb|EEC69807.1|	c83558_g1_i2	2.199	1.68E-02	4.774	4.36E-06	hypothetical protein OsI_00114 [Oryza sativa Indica Group]
gi|242061230|ref|XP_002451904.1|	c86140_g4_i1	6.894	1.41E-03	2.842	1.89E-02	hypothetical protein SORBIDRAFT_04g009750 [Sorghum bicolor]
	c86140_g4_i4	3.382	1.98E-02	5.202	3.32E-04	
gi|242082690|ref|XP_002441770.1|	c108670_g3_i2	8.228	5.19E-06	4.179	7.33E-03	hypothetical protein SORBIDRAFT_08g002056 [Sorghum bicolor]
	c87617_g7_i1	7.443	1.65E-04	7.436	1.33E-04	
gi|242082722|ref|XP_002441786.1|	c104391_g1_i1	3.805	3.77E-07	2.579	6.18E-04	hypothetical protein SORBIDRAFT_08g002290 [Sorghum bicolor]
	c104391_g1_i3	4.440	7.67E-08	2.081	5.48E-03	
	c108711_g1_i1	3.783	9.38E-07	2.252	3.43E-03	
	c81573_g3_i1	8.593	7.45E-07	4.211	5.15E-03	
	c81573_g4_i1	6.315	9.22E-03	2.583	4.09E-02	
	c88513_g2_i1	2.802	6.63E-04	4.141	1.07E-05	
gi|242082800|ref|XP_002441825.1|	c86447_g1_i2	5.341	8.23E-06	3.133	1.34E-02	hypothetical protein SORBIDRAFT_08g002950 [Sorghum bicolor]
gi|242084342|ref|XP_002442596.1|	c96572_g2_i5	1.844	9.69E-03	2.067	9.74E-03	hypothetical protein SORBIDRAFT_08g022670 [Sorghum bicolor]
gi|27542778|gb|AAO16711.1|	c104391_g1_i4	10.834	9.54E-13	5.610	1.23E-08	truncated Xa1-like protein [Sorghum bicolor]
gi|357144043|ref|XP_003573148.1|	c83558_g1_i1	3.287	1.02E-04	1.832	3.90E-02	PREDICTED: putative disease resistance protein RGA4-like [Brachypodium distachyon]
gi|414591554|tpg|DAA42125.1|	c101028_g1_i1	7.789	3.57E-05	6.349	2.15E-02	TPA: hypothetical protein ZEAMMB73_852544 [Zea mays]

### Stress responses

Several stresses can lead cell death and leaf abscission in plants, like drought [61, 62] and salt [63] stresses. Hence, we analyzed the transcripts involved in the response to stresses. Compared to LPSP, 38 transcripts were upregulated in LASP and annotated to respond the stresses (Table 5). Notably, 18 (47.37 %) transcripts of them had the ability of encoding disease resistance proteins, including 4 RGA2 (resistance gene analog 2) transcripts, 3 At1g50180 homolog transcripts and 2 RGA4 (resistance gene analog 4) transcripts. In addition, we identified 6 upregulated transcripts encoding the hypothetical protein (SORBIDRAFT_08g002290) in response to stresses. Although the function of SORBIDRAFT_08g002290 is not clear, several important motifs like leucine-rich repeats, AAA ATPase and NB-ARC domains are found in SORBIDRAFT_08g002290 [64]. It is said that proteins containing the short motif of leucine-rich repeats can regulate the interactions between proteins [65] and NB-ARC domain has been found in many resistance proteins [66]. These evidence told us that transcripts encoding proteins in response of stresses can act in the pathway of plant-pathogen interactions, and their over-expression indicated some relationship with leaf abscission in sugarcane.

**Table 6:** ABA associated transcripts up regulated in T sugarcane plants, compared to B

Gene family	gene_id	logFC(Q1/Q2)	*P*Value	logFC(T/B)	*P*Value	Description	Relationship with abscisic acid
AB2C	c53535_g1_i1	0.000	1.00E + 00	5.712	4.91E-02	ABC transporter C family member 2	abscisic acid D-glucopyranosyl ester transmembrane transport
ABAH2	c60338_g1_i1	−0.050	1.00E + 00	2.701	3.56E-02	Abscisic acid 8′-hydroxylase 2	abscisic acid catabolic process
ABAH3	c78622_g1_i1	0.307	8.54E-01	2.924	9.01E-03	Abscisic acid 8′-hydroxylase 3	
	c78622_g2_i1	−0.440	7.00E-01	2.794	1.07E-02		
AI5L3	c84393_g3_i1	−1.790	9.68E-02	3.982	5.51E-04	ABSCISIC ACID-INSENSITIVE 5-like protein 3	abscisic acid-activated signaling pathway
AMO	c101773_g1_i1	8.258	2.79E-19	3.002	3.94E-02	Primary amine oxidase	
CDPKU	c64532_g1_i2	0.000	1.00E + 00	10.382	1.56E-04	Calcium-dependent protein kinase 30	
CRK29	c100815_g2_i1	5.974	2.54E-07	4.499	1.04E-02	Cysteine-rich receptor-like protein kinase 29	response to abscisic acid
	c80509_g3_i1	0.000	1.00E + 00	7.704	3.33E-03		
FERON	c86070_g1_i3	5.144	2.25E-05	2.851	3.28E-02	Receptor-like protein kinase FERONIA	abscisic acid-activated signaling pathway
	c89220_g1_i1	−0.241	9.09E-01	2.685	2.75E-02		
GG3	c67489_g1_i3	0.000	1.00E + 00	6.142	2.91E-02	Guanine nucleotide-binding protein subunit gamma 3	response to abscisic acid
IP5P3	c103894_g2_i1	−7.938	2.25E-05	2.181	4.56E-02	Type I inositol 1,4,5-trisphosphate 5-phosphatase CVP2	abscisic acid-activated signaling pathway; response to abscisic acid
	c91503_g1_i4	−0.239	1.00E + 00	1.926	2.67E-02		
IP5PB	c95707_g1_i3	2.637	1.03E-03	2.479	1.55E-02	Type I inositol 1,4,5-trisphosphate 5-phosphatase 11	response to abscisic acid
LPP2	c101924_g4_i1	2.513	4.64E-03	1.874	2.85E-02	Lipid phosphate phosphatase 2	abscisic acid-activated signaling pathway
LTI65	c97091_g1_i2	12.717	3.19E-18	4.848	1.35E-03	Low-temperature-induced 65 kDa protein	abscisic acid-activated signaling pathway;
MFT	c87994_g2_i6	9.022	6.69E-08	5.653	2.18E-02	Protein MOTHER of FT and TF 1	response to abscisic acid
MPK5	c83394_g1_i1	4.061	5.22E-08	1.615	2.92E-02	Mitogen-activated protein kinase 5	abscisic acid-activated signaling pathway
NCED1	c102008_g2_i1	2.225	4.93E-02	3.563	2.23E-02	9-cis-epoxycarotenoid dioxygenase 1, chloroplastic	abscisic acid biosynthetic process
	c73153_g1_i1	4.064	5.24E-01	5.300	2.15E-03		
PXG4	c60264_g1_i1	5.998	2.19E-02	2.708	3.13E-02	Probable peroxygenase 4	response to abscisic acid
PYL2	c100077_g1_i1	−9.503	3.94E-09	6.381	1.09E-05	Abscisic acid receptor PYL2	abscisic acid binding;
VIV1	c127665_g1_i1	0.000	1.00E + 00	5.318	2.48E-02	Regulatory protein viviparous-1	abscisic acid-activated signaling pathway
Y1571	c97220_g1_i1	−0.460	7.64E-01	3.447	5.68E-03	Probable leucine-rich repeat receptor-like protein kinase At1g35710	response to abscisic acid
	c97220_g1_i2	−1.118	2.71E-01	3.092	2.27E-02		

### ABA-associated transcripts

In the abscission process, ABA plays a critical role and involves in different pathways [67]. In citrus, leaf abscission induced by rehydration after a period of water stress requires ABA accumulation [62]. In our study, we found different upregulated transcripts involved in ABA signaling pathways in the two sugarcane generations. Compared to Q2 and B, a total of 341 and 26 upregulated ABA-associated transcripts were identified Q1 (Additional file 6) and T (Table 6), respectively. Q1 and T shared 10 upregulated transcripts involved in ABA signaling pathways. GO annotation showed commonly upregulated transcripts were involved in ABA D-glucopyranosyl ester transmembrane transport, ABA catabolic process, ABA-activated signaling pathway, response to ABA and ABA binding. Compared to T sugarcane, Q1 had more upregulated ABA-associated transcripts, like ABC (ATP-binding cassette) transporter G family members (5, 11, 15, 25, 36, and 40), ABA receptor (PYL2 and PYL8) and zeaxanthin epoxidase (ZEP). In *Arabidopsis*, ABA transport can be mediated by PDR-type ABC transporter, ABG25 and AB40G [68, 69]. ZEP also plays an important role in the xanthophyll cycle and ABA biosynthesis, can convert zeaxanthin into antheraxanthin and subsequently violaxanthin, and is required for resistance to osmotic and drought stresses, seed development and dormancy [70].

### Transcription factors

Further, 2,031 of the assembled transcripts were annotated to encode TFs in this study. There were 603 and 58 transcripts were upregulated in Q1 and T relatively to Q2 and B, respectively. Of the upregulated transcripts encoding TFs, 16 were in common, including two transcripts encoding MADS-box transcription factors, six transcripts encoding heat stress transcription factors, two transcripts encoding transcription factor DIVARICATA, one WRKY33 transcript, two transcripts encoding NAC transcription factor 29, and three transcripts encoding ethylene-responsive transcription factor. Although the function of these TFs in sugarcane is not clear, they have been reported to regulate the leaf senescence and abscission in Arabidopsis [71–73].

Overall, pathways of plant-pathogen interaction, response to stresses and ABA signaling are important pathways in plants and involve in several bio-activities including cell development and death [50, 74]. Although it is hard to tell which pathways are involved in the regulation of leaf abscission in sugarcane, the up regulation of transcripts involved in these pathways strongly supports that they may play an important role in the AZ development and leaf abscission in sugarcane. Q1 and T upregulated transcripts were enriched in different pathways, we can infer that they may promote the shedding of their leaves in different ways during the maturity time. Transcripts identified here showed their potential availability which can be used in sugarcane breeding programs.

### qRT-PCR validation

**Fig. 4:**
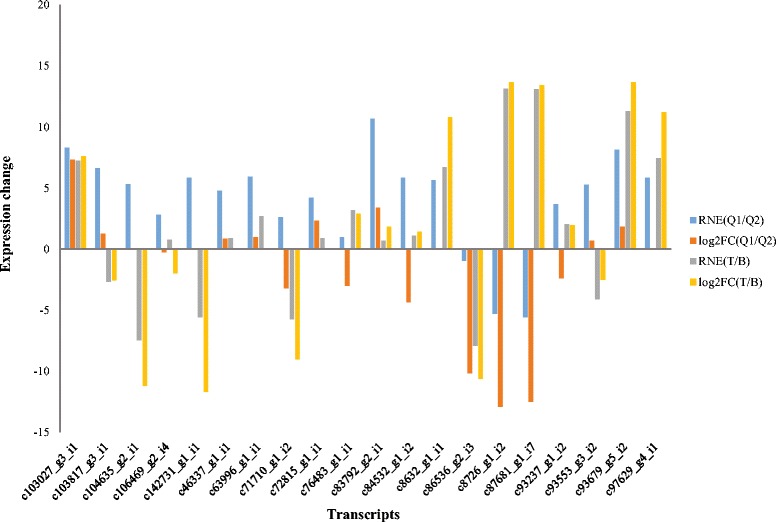
qRT-PCR validation for candidate transcripts

Differentially expressed transcripts between LASP and LPSP identified by RNA-Seq were confirmed by quantitative real-time PCR (qRT-PCR) experiment. A total of 20 transcripts were randomly selected as candidates. We used RT^2^ First Strand Kit (QIAGEN) and PCR mix (QIAGEN) to perform the cDNA synthesis and real-time PCR experiment. Forward and reverse primers for these 20 candidate transcripts were designed by Primer Express Software (v2.0, ABI) and actin gene was used as the reference gene. The information of primers used in this study can be found in Additional file 7. As shown in Fig. 4, we used relatively normalized expression (RNE) and log 2 fold change (log2FC) to show the expression changes of candidate transcripts in Q1 vs. Q2 and T vs. B identified by qRT-PCR and RNA-Seq, respectively. The dysregulation of 19 transcripts (90.48 %) were consistent between qRT-PCR and RNA-Seq. Especially for those transcripts absent in several samples, they were not detected by both qRT-PCR and RNA-Seq. The Pearson correlations showed high relevance between RNA-Seq and qRT-PCR (0.911 for Q1/Q2 and 0.971 for T/B).

### SNP discovery

**Fig. 5:**
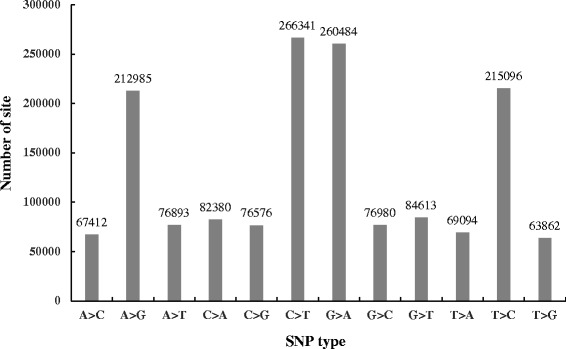
Different types of SNPs identified in this study

Specificity and high abundance of genes support we can call single nucleotide polymorphisms (SNPs) using RNA-Seq [75]. RNA-Seq has been used for SNP discovery in non-model plants such as *Eucalyptus grandis* [76], *Brassica napus* [77], and *Medicago sativa* [78]. In this study, we used samtools [79] and bcftools to call SNPs, and found a total of 1,544,787 SNPs in the transcriptome alignment files for six libraries. The numbers of different SNP types were shown in Fig. 5. Because multiple SNP types could happen at one site, the total number of SNP types is a little greater than the total SNP site number (1,544,787). A total of 285,080 SNPs (18.45 %) were annotated to occur in the potential protein coding regions. Four SNP types (A- > G, C- > T, G- > A and T- > C) were significant because they took 61.50 % of the total SNP types in this study. Finally, we identified 3,722 SNPs, which were different between LASP and LPSP but same in LASP or LPSP. As shown in Additional file 8, these 3,722 SNPs were divided into 1,134 nonsynonymous SNPs and 2,588 synonymous SNPs. KEGG pathway analysis showed the transcripts containing nonsynonymous SNPs were enriched in the pathways of cytosolic DNA-sensing pathway, ribosome biogenesis in eukaryotes and inositol phosphate metabolism. Due to limit sugarcane gene annotation, SNP results seemed not relevant to our aim, which is simply to identify leaf abscission associated genes in sugarcane, more experiments and data are required to investigate the functions of these SNPs in leaf abscission.

## Conclusions

In current study we employed next generation sequencing method RNA-Seq to analyze the transcriptome of sugarcane leaves and characterize candidate genes related to the leaf abscission in sugarcane during the maturity time. A total of 215,019,578 transcripts were initially assembled with N50 of 1,177 bp in length. We annotated them by mapping them to several known databases such as NR, UniProtKB/Swiss-Prot, GO and KEGG pathway databases. Further annotations including signal protein, rRNAs, protein domains and membrane proteins were performed by Transdecoder and Trinotate pipelines. Based on the assembled transcriptome, we identified several transcripts differentially expressed between LASP and LPSP, which were annotated to involve in the plant-pathogen interaction, stress response and ABA-associated pathways. qRT-PCR was used to validate the expression levels of 20 randomly selected transcripts. The results showed high consistency between qRT-PCR and RNA-Seq methods. Furthermore, a total of 1,544,787 SNPs were identified successfully, of them 1,134 were nonsynonymous SNPs and 2,588 were synonymous SNPs. The transcriptome produced by this study will provide a valuable resource of molecular information for future investigations and process understanding the roles of genes in the leaf abscission of sugarcane. Our study may also help develop sugarcane varieties which may shed their leaves during the maturity time and benefit the sugarcane farmers.

## Methods

### Plant material

The sugarcane plants were grown and maintained in the experimental filed of Sugarcane Research Institute in Nanning, Guangxi Province of China. ROC-26 (from Taiwan) is a precocious and productive sugarcane variety, but the sugar cane is wrapped very tight at the physiological maturity [80]. GT96-167 is a late-maturing and high-yield sugarcane variety which is bred by Guangxi Sugarcane Research Institute [81–83]. In contrast with ROC-26, GT96-197 can shed their leaves easily during the maturity time. By using 19 GT96-167 and 12 ROC-26 sugarcane plants, we obtained 83 pairs of sugarcane sexual hybridizations. For each hybridization, 3–6 g seeds (germination number: 30-260/g) were used to cultivate a total of 34,000 sugarcane seedlings. After 4 years, 49 hybridizations were proved to have distinct phenotypes on leaf shedding among their F1 generations. In this study, we chose two F1 generation sugarcane varieties 42–1 and 42–2 (named as T1 and T2) which can shed their leaves during the maturity time and two another F1 generation sugarcane varieties 42–6 and 42–16 (named as B1 and B2) which cannot shed their leaves during the maturity time, as well as their parents GT96-167 and ROC-26 (named as Q1 and Q2). According to the cultivation and sugar content test, sugarcane harvest time is always in the period of mid-November to the next mid-March in China. Six sugarcane plants (Q1, Q2, T1, T2, B1 and B2) were planted in January of 2014. And on 24^th^ December of 2014 we performed the sugar content test for all six sugarcane plants and found the sugar content of each plant reached (or was close to) the peak. New born leaf tissues approximately 5 cm above the growing point were taken on 5^th^ December of 2014. The leaf tissues were stored in the liquid nitrogen before RNA extraction. The F1 generation sugarcane varieties were proved to have stable agronomic characteristic on leaf shedding by 5 years of filed observation.

### RNA extraction

Total RNA was isolated from sugarcane leaves by using TRIzol® reagent (Invitrogen) according to the manufacture’s protocol. Briefly, 1 ml of TRIzol® reagent was added into 100 mg of leaf sample, the sample was homogenized using power homogenizer and centrifuged at 12,000 × g for 10 min at 4 °C. After the fatty layer was removed and discarded, the cleared supernatant was transferred into a new tube and mixed with 0.2 ml of chloroform. The tube was shaken vigorously for 15 s, followed by an incubation for 3 min at room temperature. Next, the sample was centrifuged at 12,000 × g for 15 min at 4 °C and the aqueous phase was moved into a new tube for RNA precipitation. For precipitating RNA from each sample, 10 μg of RNase-free glycogen was added into the aqueous phase as a carrier, followed by 0.5 ml of 100 % isopropanol, then, samples were incubated at room temperature for 10 min and centrifuged at 12,000 × g for 10 min at 4 °C. To wash the RNA pellet, we added 1 ml of 75 % ethanol into the tube, vortexed the tube gently, centrifuged the tube 7,500 × g for 5 min at 4 °C and discarded the wash. The RNA pellet was air-dried, suspended in RNase-free water, water bathed at 60 °C for 10 min, quality controlled by Agilent 2100 Bioanalyzer and used for cDNA library construction and deep sequencing.

### cDNA library construction and transcriptome sequencing

Equal amount of total RNA (20 μg) was used for cDNA library construction using TruSeq™ RNA Sample Preparation Kit v2 (Illumina) and for transcriptome sequencing on the Illumina HiSeq 2000 platform, according to protocols. Briefly, total RNA was used to isolate poly(A) mRNAs using Dynal Oligo(dT) beads (Invitrogen). Then, mRNAs were chemically fragmented to ~200 nt fragments by using divalent cations (Elute/Prime/Fragment Mix buffer, Illumina) under elevated temperature. Cleaved RNA fragments were then copied into first strand cDNA by using reverse transcriptase and random primers, followed by the second strand cDNA synthesis using DNA Polymerase I (Invitrogen) and RNase H (Invitrogen) treatment. The cDNA fragments were next end repaired by using End Repair Mix (Illumina) reagent, purified and enriched with PCR to create the final cDNA libraries. Six cDNA libraries were sequenced by using pair-end (2 × 90 bp) sequencing technology with an Illumina HiSeq™ 2000 sequencer. The sequencing raw data (FASTQ formatted files) for six samples can be accessed in the NCBI Sequence Read Archive (SRA) database (http://trace.ncbi.nlm.nih.gov/Traces/sra/) under the accession number of SRA291189.

### De novo assembly of the transcriptome

Raw reads of six libraries were cleaned by removing adapter sequences and low-quality sequences (reads with ambiguous bases ‘N’ and reads with more than 10 % Q < 20 bases). The resulting high quality reads of each library were quality controlled by using the program FastQC (http://www.bioinformatics.babraham.ac.uk/projects/fastqc/) before assembly. De novo assembly of the transcriptome was performed by Trinity software (release 2014-07-17) [30], according to the protocol [84]. Initially, highly quality RNA-Seq reads were used to generate overlapping k-mers (25). Based on the (k-1)-mer overlaps, Inchworm was used to assemble sorted k-mers into transcript contigs. Next, Chrysalis was used to cluster related Inchworm contigs into components by using grouped raw reads and paired read links. Then, a de Bruijn graph for each cluster was built by Chrysalis and reads were partitioned among the clusters. Finally, Butterfly was used to process the individual graphs and ultimately report the full length transcripts. To ensure a uniform transcriptome reference across samples, all clean reads were pooled together for assembly, then clean reads of six samples were individually aligned back to the assembled transcriptome reference.

### Extract likely coding sequences from Trinity transcripts

We used TransDecoder, which is included in the Trinity software distribution, to extract potential coding sequences from assembled transcripts. Briefly, the longest open reading frame (ORF) was first identified within the assembled transcripts. Using a Markov model based on hexamers, a subset corresponding to the very longest transcripts were identified from all the longest ORFs. Then, all the longest ORFs identified were scored according to the Markov Model (log likelihood ratio based on coding/noncoding potential) in each of the six possible reading frames. For a particular transcript, the ORF was reported when its proper coding frame score calculated by GeneID software [85] was positive and highest of the other presumed wrong reading frames. A high scored putative ORF was excluded when it was fully encapsulated by the coordinates of another candidate ORF. The identified ORFs were set to encode a protein at least 100 amino acids.

### Functional annotation of assembled transcripts using Trinotate

After the ORFs were extracted from the assembled transcripts, the deduced proteins were annotated using Trinotate (r2014-07-08, available at http://trinotate.github.io/). Briefly, deduced proteins were used to search against UniProtKB/Swiss-Prot database (ftp://ftp.broadinstitute.org/pub/Trinity/Trinotate_v2.0_RESOURCES/uniprot_sprot.trinotate_v2.0.pep.gz) to identify known protein sequences. Functional domains were identified by mapping them to the PFAM domain database (ftp://ftp.broadinstitute.org/pub/Trinity/Trinotate_v2.0_RESOURCES/Pfam-A.hmm.gz) [86] using HMMER [87]. Potential signal peptides, transmembrane domains and rRNA transcripts were predicted by SignalP [36], TMHMM Sever v2.0 [37] and RNAMMER [88], respectively. Then, the likely protein sequences were used to search against EggNOG database (v4.1, http://eggnogdb.embl.de) [38] which enables to identify the proteins distributed in EuKaryotic Orthologous Groups (KOG), Clusters of Orthologous Groups (COGs), and non-supervised orthologous groups (NOGs). Finally, above annotations were loaded into a Trinotate SQLite database and a final annotation report was generated. The maximum e-values for reporting best hit and associated annotation were no more than 1e-5.

### GO and KEGG pathway annotation

To identify the assembled transcripts related with Gene Ontology (GO) and biology pathways, they were annotated by comparing to previously annotated genes in three public databases, NCBI non-redundant (NR) database, Swiss-Prot database and Kyoto Encyclopedia of Genes and Genomes (KEGG) database. We used BLAST software [89] to map assembled transcripts against NR database and filtered the mapping hits using a cut-off of e-value (1 × 10^−5^). The resulting transcripts were then processed to retrieve associated Gene Ontology (GO) items describing biological processes (BPs), cellular components (CCs) and molecular functions (MFs) by BLAST2GO software [90]. By using unique gene accession numbers, BLAST2GO also produces corresponding enzyme commission (EC) numbers for sequences with an e-value ≤1e-5. Then, the transcripts with corresponding ECs were obtained and mapped to KEGG metabolic pathway database.

### Transcriptome profile and different expression

The abundance of each transcript was evaluated by Bowtie2 and RSEM (RNA-Seq by Expectation-Maximization) tools in every sample. First, high quality reads of each library were mapped to Trinity assembled transcripts by Bowtie2 [41]. Then, an R package RSEM was used to evaluate the expression levels of each transcript in every library by estimating the abundance of reads that aligned to the transcript. Differential expression of transcripts across samples was identified by using an R package called edgeR [43]. edgeR can proceed differential expression of a transcript in two groups as we performed biological replicates for T and B sugarcane varieties in this study. The significance of differential expression was evaluated by the fold change (≥2) and *p*-value (<0.05).

### SNP discovery

As the phenotypes of sugarcanes used in this study were different, we used samtools (v1.2, https://github.com/samtools/samtools/releases/download/1.2/samtools-1.2.tar.bz2) [79] and bcftools (v1.2, https://github.com/samtools/bcftools/releases/download/1.2/bcftools-1.2.tar.bz2) to find possible single nucleic polymorphisms (SNPs). In brief, clean reads of each sample were aligned against the assembled transcriptome reference by Bowtie2, generating BAM formatted files. The BAM files were then indexed and processed by the mplieup function of samtools to produce a BCF file that contains all the locations in the genome. The BCF file was then used to call genotypes and reduce the list of sites to those found to be variant by passing this file into bcftools call. Finally, after filtering low quality SNPs, reliable SNPs were left and stated by a self- developed Perl script.

### GO and KEGG pathway enrichment analysis

Functional analysis was performed using the Gene Ontology and KEGG pathway annotations for the transcripts. To find enriched GO items and KEGG pathways, we used *p-value* (Fisher’s exact test) and *q- value* [91] to show the significance of enrichment and control the false discovery rate. Significant GO items and KEGG pathways should satisfy the critical of *p*-value < 0.05 and q-value < 0.9. Detected pathways only related to animal or human GO items and KEGG pathways were filtered.

### Quantitative real-time PCR

Quantitative real-time PCR (qRT-PCR) experiment was used to validate the expression patterns of RNA transcripts in different sugarcane varieties. Total RNA (4 μg) which was used for RNA-Seq previously described was used for cDNA synthesis by using RT^2^ First Strand Kit (QIAGEN) and qRT-PCR was performed by using PCR mix (QIAGEN), according to the manufactures’ protocols. Briefly, genomic DNA elimination mix (10 μl) was first made by using total RNA (4 μg), Buffer GE and RNase-free water. After incubated at 42 °C for 5 min and on ice for 5 min, the genomic DNA elimination mix was mixed with 5x Buffer BC3 (4 μl), control P2 (1 μl), RE3 Reverse Transcriptase Mix (2 μl) and RNase-free water (3 μl). The final reverse transcription mix (20 μl) was incubated at 42 °C for exactly 15 min and at 95 °C for 5 min to finish cDNA synthesis. Primers for qRT-PCR were designed by Primer Express Software (v2.0, ABI) and synthesized by BGI (Additional file 7). A total of 16 μl reaction mix was made by 2x PCR mix (8 μl), forward primer (0.2 μl, 50pM/ul), reverse primer (0.2 μl, 50pM/ul), cDNA template (1 μl) and RNase-free water (6.6 μl). The final cDNA concentration of each reaction was 12.5 ng/μl. Actin was used as control and three reactions were performed for each transcript in every sample. The PCR reaction was performed and analyzed by using ABI ViiA 7 Real Time PCR System. After the threshold cycle (C_T_) numbers of each transcript in every samples were evaluated, mean C_T_ values were calculated for subsequent analysis. Base on the mean C_T_ value, ΔC_T_ was used to present and normalize the expression of a candidate transcript. Relatively normalized expression (RNE, −ΔΔC_T_ method) was used to show the expression change of a transcript in two samples. C_T_ values greater than 35 were set to 35.

## Additional files

Additional file 1: Number of sugarcane transcripts aligned to different species. (XLSX 19 kb) Additional file 2: Gene Ontology annotation for the assembled transcriptome of sugarcane leaves. (TXT 3759 kb) Additional file 3: Commonly upregulated transcripts in Q1/Q2 and T/B. (XLSX 320 kb) Additional file 4: KEGG pathway analysis for Q1 sugarcane upregulated transcripts relatively to Q2 sugarcane. (XLSX 14 kb) Additional file 5: KEGG pathway analysis for T sugarcane upregulated transcripts relatively to B sugarcane. (XLSX 11 kb) Additional file 6: ABA-associated transcripts upregulated in Q2 in comparison of Q1. (XLSX 38 kb) Additional file 7: Primer sequences used in qRT-PCR experiment. (XLSX 10 kb) Additional file 8: 1,134 nonsynonymous SNPs and 2,588 synonymous SNPs identified in this study. (XLSX 404 kb)

## Abbreviations

ABA: abscisic acid
ABC: ATP-binding cassette
AZ: abscission zone
COG: Clusters of Orthologous Group
EC: enzyme commission
GO: gene ontology
KEGG: Kyoto Encyclopedia of Genes and Genomes
KOG: EuKaryotic Orthologous Group
LASP: leaf abscission sugarcane plants
LPSP: leaf packaging sugarcane plants
NOG: non-supervised orthologous group
ORF: open reading frame
qRT-PCR: quantitative real-time polymerase chain reaction
RIN4: RPM1-interacting protein 4
RPM1: resistance to Pseudomonas syringae pv. Maculicola 1
RPP13: recognition of Peronospora parasitica 13
RSEM: RNA-Seq by Expectation-Maximization
SNP: single nucleotide polymorphism
SRA: sequence read archive
TF: transcription factor
ZEP: zeaxanthin epoxidase
